# Leveraging the perfusionist-surgeon dyad to improve the culture of safety

**DOI:** 10.1051/ject/2025022

**Published:** 2025-12-17

**Authors:** Kenneth G. Shann, Thoralf M. Sundt

**Affiliations:** 1 Division of Cardiac Surgery, Mass General Brigham Boston MA USA

**Keywords:** Cardiac surgery, Culture, Teamwork, Surgeon, Perfusionist

## Abstract

The cardiac surgical operating room is a complex, high risk environment dependent on efficient teamwork and communication between multiple role groups to deliver safe and effective care. As awareness of the critical importance of culture on team performance broadens, there has been increasing focus in cardiac surgery on developing a culture of safety to minimize, trap and recover from errors in order to optimize patient outcome. Fundamental to this effort are concepts such as Just Culture, collective responsibility and perhaps most fundamentally the establishment of psychological safety where individuals have a “sense of being able to show and employ oneself without fear of negative consequences to self-image, status or career”. Team members who are engaged and feel empowered to raise concerns can help to recognize issues, improve intraoperative decision making and contribute to resilience of the team. Over the past decade we have made a concerted effort in our institution to establish such a culture, and have found principles of organizational psychology helpful in this endeavor.

## Overview

The cardiac surgical operating room is a complex, high-risk environment dependent on efficient teamwork and communication between multiple role groups to deliver safe and effective care [1]. As awareness of the critical importance of culture on team performance broadens, there has been increasing focus in cardiac surgery on developing a culture of safety to minimize, trap, and recover from errors in order to optimize patient outcomes. Fundamental to this effort are concepts such as Just Culture, collective responsibility and perhaps most fundamentally the establishment of psychological safety where individuals have a “sense of being able to show and employ oneself without fear of negative consequences to self-image, status or career” [2]. Team members who are engaged and feel empowered to raise concerns can help to recognize issues, improve intraoperative decision making and contribute to resilience of the team [3]. Over the past decade, we have made a concerted effort in our institution to establish such a culture, and have found principles of organizational psychology helpful in this endeavor.

Traditionally, operating room teams have operated under an extreme professional hierarchy that inhibits psychological safety, where those in higher positions of authority, i.e., surgeon leaders, have increased freedom to speak relative to team members in lower positions on the hierarchy [4]. This can prevent individuals in lower positions from speaking up and expressing concerns, which can lead to missed opportunities for error prevention, improved decision making, and learning [5]. This was profoundly true in our organization historically, which led to difficulty in recruiting and retaining staff among multiple role groups in the operating room. While some hierarchy is necessary and appropriate, leaders can practice behaviors of inclusiveness that maintain efficient and effective decision-making while also engaging other team members, i.e., perfusionists, for their thoughts and ideas [4]. This is particularly pertinent as operative procedures have become more complex and longer in duration, leading to increased cognitive load demands on the surgeon, potentially exceeding safe thresholds and impacting performance [6]. To mitigate this risk, it would be desirable for team members, i.e., perfusionists, anesthesiologists, nurses, to have a shared mental model of the surgical plan and speak up when there are unintended deviations from that plan. Studies have demonstrated that there can, in fact, be wide variation in shared mental models within and between role groups caring for cardiac surgical patients [7].

The perfusionist-surgeon dyad is a unique relationship that has evolved over the 70-plus years since the introduction of extracorporeal circulatory support. Unlike other role group relationships, such as those among nurses, surgeons, and anesthesiologists which exist in some form or another broadly throughout the operating room, perfusion support (at least traditionally) was confined to the cardiac surgery operating room. While the perfusionist-anesthesiologist dyad is critically important, the most intimate relationship is with the surgeon, with whom real-time problem-solving must occur over critical patient care issues such as flow rates, drainage, temperature management, and myocardial preservation. In many institutions, the perfusion team feels more tightly connected to the cardiac surgeons than other members of the OR staff, and vice versa. Finding ourselves in positions of organizational authority and believing that addressing these cultural issues was foundational to the long-term success of our unit, we worked together, leveraging the unique relationship that exists between perfusionists and surgeons to effect the change we sought to achieve throughout the operating room. The impact has been powerful, extending beyond the perfusionist-surgeon axis to effectively create an environment of teamwork and communication among all caregivers in the cardiac surgical operating room.

## Description

**Figure 1:**
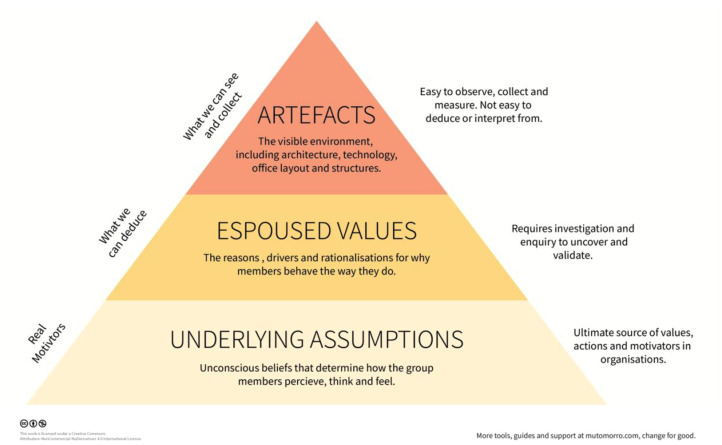
Edgar Schein’s Culture Model.

Committed to effecting culture change, we borrowed concepts from the business world. In understanding Organizational Culture, Edgar Schein describes three components of organizational culture, or “levels” on which the culture is manifest: artifacts, espoused values, and underlying assumptions [8] (Figure 1) Artifacts are the visible attributes, easily observed by outsiders, such as the hospital logo, colors, and slogans. Values are the stated beliefs and goals that the organization publicly communicates through mission statements, websites, and employee training. The deepest layer of culture is the underlying assumptions, and include the unconscious beliefs and perceptions people within the organization hold to be true but have never challenged, tested, or verified. Examples of assumptions in our context might include accepting unfavorable behavior from a surgeon because “all cardiac surgeons are like that” or believing that our current practice already delivers the best possible quality and safety or that optimal teamwork exists simply because we are who we are (we believe our own press). Often, underlying assumptions are formed through years of individuals working together and assuming there will never be a different or better way. Accordingly, those within the culture struggle to even imagine asking the question.

Twelve years ago, the leadership of the Division of Cardiac Surgery at Massachusetts General Hospital (MGH) committed to the defined goal of “making MGH the best place in the world to learn and practice cardiac surgery”. To achieve that goal, two significant changes would need to occur: 1) develop an environment of teamwork characterized by psychological safety for all members, and 2) challenge the underlying assumptions that had existed for decades and defined the local culture in cardiac surgery. As one of the most successful academic institutions in the world with a rich history of high-quality care, teaching, and research, broadly speaking, MGH was a fertile ground to do this work. However, work needed to be done within the cardiac surgery unit. The hierarchy in favor of the surgeon was stiff, intimidating, and unquestioned. Staff turnover was high among many role groups, the cardiac surgical residency struggled to attract applicants, and the perfusion team was not structured to support significant growth and change. We elected to employ a strategy to change culture by clearly stating (espousing) the Values on which the service would rest, and by instituting Artifacts deliberately intended to send a clear message regarding expected behaviors that facilitate teamwork and communication.

We began by establishing among the surgical staff that we would: 1) put the needs of patients first, 2) make a contribution to the field through research, education and innovation, and 3) recognize that the success of each individual depends on the success of the group as a whole and the success of the group depended on the success of each individual. The first was adopted from the Mayo Clinic, the second made clear why we were practicing at MGH rather than in private practice, and the third addressed most clearly how we would function together as a team, supporting each other and supporting the collective group. In perfusion, we first developed a mission statement to act as a beacon and reminder of where we were going:

“The Perfusion Team at Massachusetts General Hospital is dedicated to providing the most innovative and patient-centered care for patients undergoing extracorporeal support. In a professional and fiscally responsible manner, the perfusionists embrace evidence-based practice, academic excellence and patient safety in order to achieve the highest quality outcomes.”

In addition, we explicitly challenged the existing underlying assumptions:

- “We are going to recruit the best and brightest individuals to join our team, including surgeons, anesthesiologists, intensivists, perfusionists, nurses, and advanced practice providers.”
- “The way we’ve always done things is not necessarily the way we’ll do them in the future.”
- “We will no longer accept variation in practice simply based on individual’s preference.”
- “Each discipline is expected to be the expert in what they do, i.e., perfusionists are experts in cardiopulmonary bypass.”
- “You are not only encouraged but expected to speak up if you are concerned.”

Over the next several years, we established Artifacts supporting an attitude of professionalism among all role groups, including implementing guidelines via a structured multidisciplinary process involving surgery, anesthesia, and perfusion. We began with the development of clinical practice guidelines for cardiopulmonary bypass (CPB), describing in detail how CPB would be managed for the majority of our patients in the majority of clinical scenarios. The guidelines were drafted by the perfusionists, reviewed and revised by the attending anesthesiologists, and ultimately reviewed, revised, and approved by all attending surgeons. As recommended by AmSECT’s Standards and Guidelines [9] the guidelines are reviewed, revised, and approved annually by all three disciplines and essentially have formed the foundation of the two dyads in our cardiac operating rooms, surgeon-perfusionist and anesthesiologist-perfusionist. The guidelines explicitly state expectations for the management of CPB:

- “The Perfusionist is expected to “Speak Up” if they have any concerns regarding patient care.”
- “The “*n* + 1” staffing model will be utilized at all times.”
- “The pre-CPB checklist will be completed by two perfusionists in a read-verify manner.”
- “Maintain indexed oxygen delivery (iDO_2_) ≥280 mL/min/m^2^ at temperatures >32 °C”
- “The anesthesiologist and/or surgeon will be consulted if the phenylephrine infusion rate requirements exceed 100 μg/min.”

**Figure 2:**
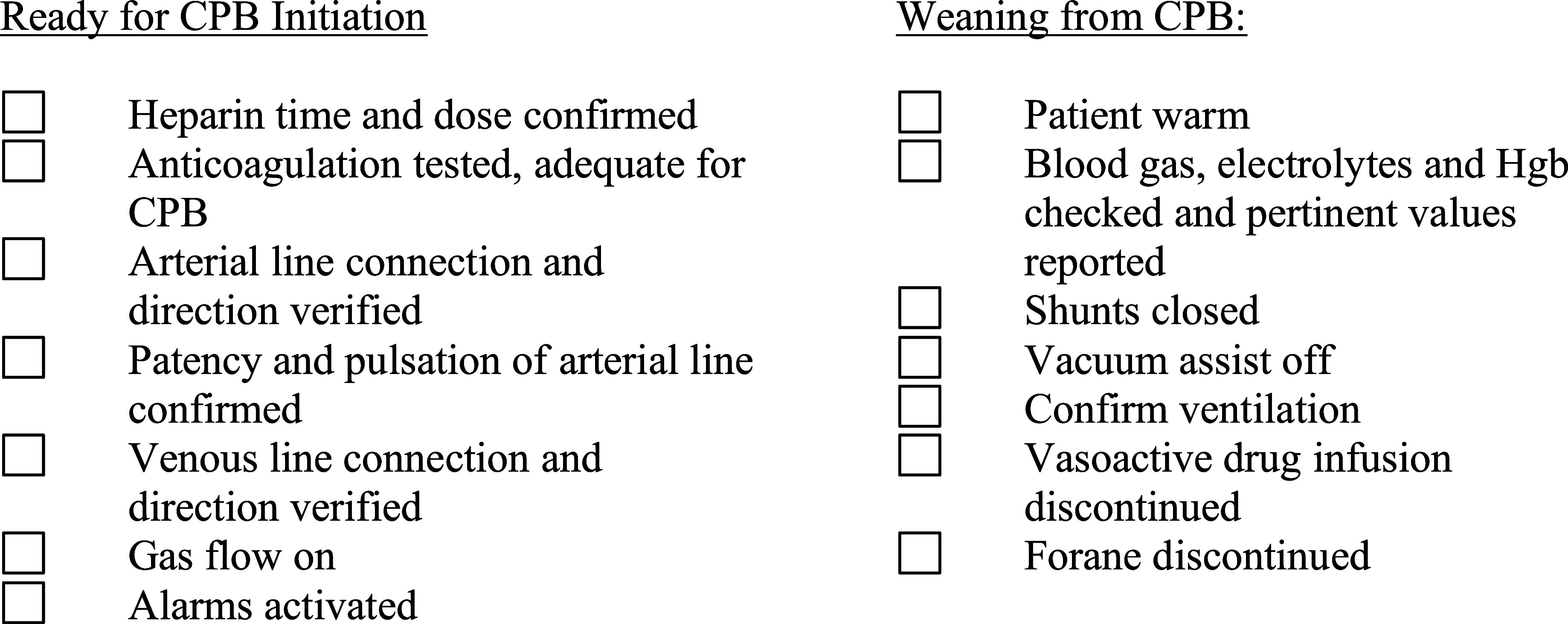
Formal checklists for the initiation and weaning of CPB.

Shortly after the approval of the CPB Guidelines, we implemented formal checklists for the initiation and weaning of CPB (Figure 2). Uniquely, these checklists are performed verbally by the perfusionist at the request of the attending surgeon prior to initiating and weaning from CPB and include elements engaging the anesthesia team. These checklists facilitated a pause in the operation to ensure all required tasks had been completed, but equally important, provided an opportunity for interdisciplinary communication regarding patient care and the establishment of situational awareness. Recently, the European multi-societal CPB Guidelines were published and included recommendations for the use of initiation and weaning checklists [10].

**Figure 3:**
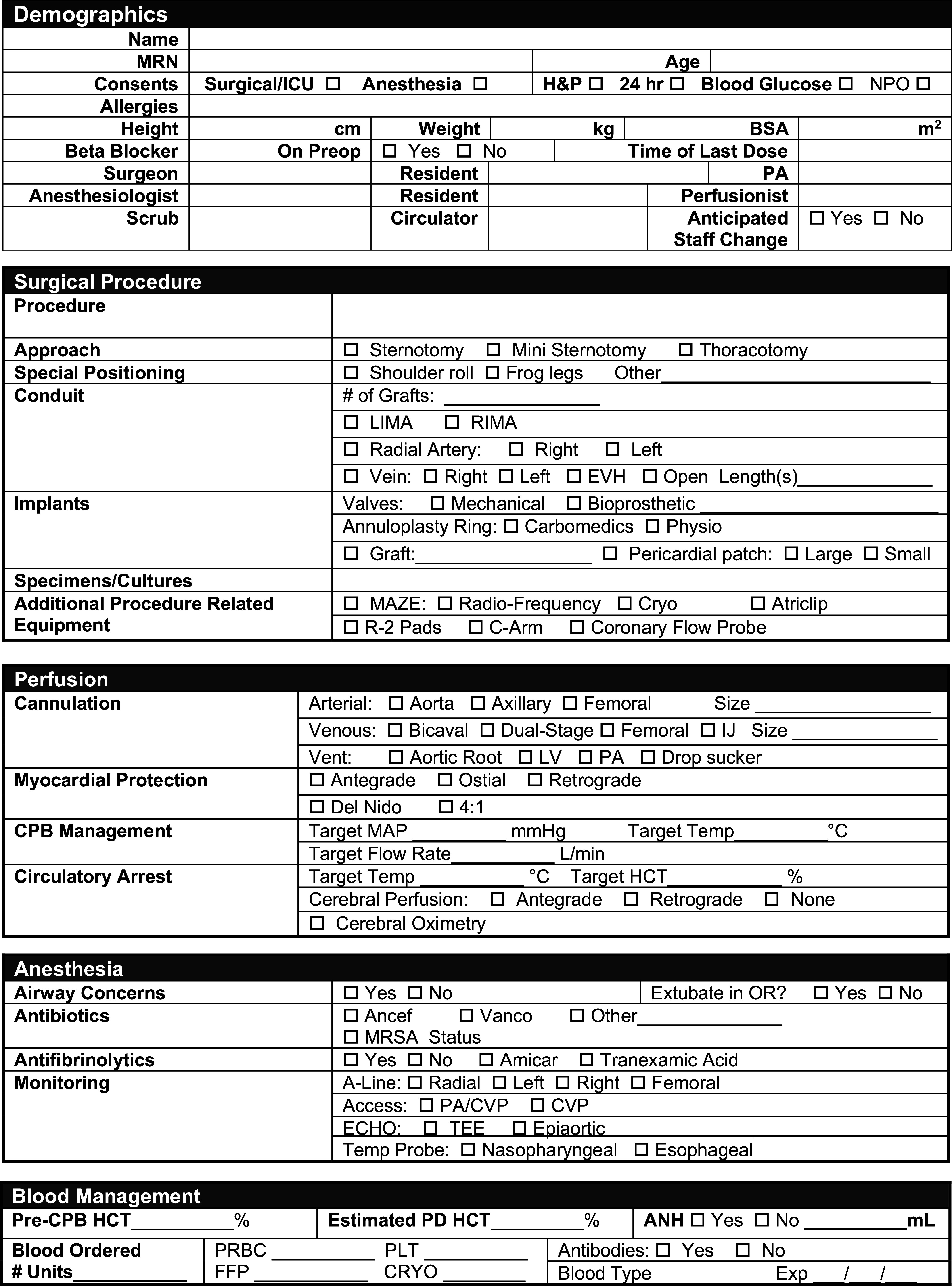
Cardiac Surgery Preoperative Briefing Form.

Our following structural change was the implementation of a formal preoperative briefing for every cardiac surgical procedure, as has been demonstrated elsewhere to have a positive impact on the team [11]. The briefing differs from the checklist in that it is multidisciplinary and participatory, with members of each role group expected to verbalize their components, thereby establishing dialogue from the beginning of the case to develop a shared mental model by allowing for questions and clarifications. Unlike a checklist, which is fundamentally the same for every patient, the briefing is different for every patient, creating an opportunity to discuss how care may be modified from routine for specific procedural or patient characteristics. The briefing form (Figure 3) hangs on the wall in each of our operating rooms and is divided into sections (surgical plan, perfusion plan, anesthesia plan, and blood management plan) and is led by the circulating nurse assigned to the case. The briefing exercise reinforces the expectation that team members will to speak up if they have any concerns during the procedure.

## Discussion

The impact of this program has been positive and profound and has extended beyond the surgeons and perfusionists to all members of the team. Anesthesia, which as a service struggled to attract residents to cardiac surgery because of the unpleasant environment in the cardiac surgical ORs, is now highly competitive for the most qualified trainees. Although we are still rebounding from the effects of the pandemic, we are more effectively attracting nursing and scrub tech staff to our environment. We have successfully recruited some of the best perfusion students from some of the best perfusion schools, i.e., MUSC, SUNY, Midwestern, and Quinnipiac, who completed their training at MGH and wanted to begin their career with our team. The cardiac surgery residency had developed a reputation for unpleasantness, with few trainees in the institution’s general surgery program wishing to remain for cardiac surgical training and had recently been unable to fill through the national match. The program is now highly competitive and frequently fills internally. The program has also now attracted the attention of other surgical groups who come to observe our practices as an example of a high-reliability unit.

Our experience indicates to us that one can effect positive culture change via consistent leadership from the perfusionist-surgeon dyad and a deliberate approach to establishing clear, explicit values and creating artifacts that support the desired culture. Given institutional will, this model should be reproducible and, we believe, will improve patient outcomes in Cardiac Surgery.

## Data Availability

The research data are available on request from the authors.
